# LncRNA RP11-670E13.6, interacted with hnRNPH, delays cellular senescence by sponging microRNA-663a in UVB damaged dermal fibroblasts

**DOI:** 10.18632/aging.102159

**Published:** 2019-08-23

**Authors:** Mengna Li, Li Li, Xiaofeng Zhang, Huijuan Zhao, Min Wei, Wanying Zhai, Baoxi Wang, Yan Yan

**Affiliations:** 1 Department of Dermatology, Plastic Surgery Hospital, Chinese Academy of Medical Sciences and Peking Union Medical College, Beijing 100144, China

**Keywords:** cellular senescence, ultraviolet B, lncRNA, microRNA, dermal fibroblast

## Abstract

Ultraviolet (UV) irradiation from the sunlight is a major etiologic factor for premature skin aging. Long noncoding RNAs (lncRNAs) are involved in various biological processes, and their roles in UV irradiation-induced skin aging have recently been described. Previously, we found that the lncRNA *RP11-670E13.6* was up-regulated and delayed cellular senescence in UVB-irradiated primary human dermal fibroblasts. Here, we performed further investigations of *RP11-670E13.6* function. The results showed that this lncRNA directly bound to *miR-663a* and functioned as a sponge for *miR-663a* to modulate the derepression of Cdk4 and Cdk6, thereby delaying cellular senescence during UV irradiation-induced skin photoaging. Moreover, we found that *RP11-670E13.6* may facilitate DNA damage repair by increasing ATM and γH2A.X levels. In addition, heterogeneous nuclear ribonucleoprotein H physically interacted with *RP11-670E13.6* and blocked its expression. Collectively, our results suggested that the *RP11-670E13.6/miR-663a*/*CDK4* and *RP11-670E13.6/miR-663a*/*CDK6* axis, which may function as competitive endogenous RNA networks, played important roles in UVB-induced cellular senescence.

## INTRODUCTION

The aging of human skin is caused by genetic and environmental factors. Among environmental factors, solar ultraviolet (UV) B (290–320 nm) and UVA irradiation (320–400 nm) are the main factors, causing atrophy of the skin, coarse wrinkles and leathery skin [1–3]. DNA photodamage and UV-generated reactive oxygen species (ROS) are the initial molecular events that lead to most of the typical histological and clinical manifestations of skin aging. [4–6]. Most DNA damage is repaired by functional repair systems in cells, once unrepairable and extensive DNA damage occurs, cells terminate proper division and enter a cell-senescent state [7]. Although numerous factors are involved in cellular senescence, the p53-p21 and p16^*CDKN2A*^ (p16)–phosphorylated retinoblastoma protein pathways are best documented in maintaining cellular senescence and growth arrest [8].

Long noncoding RNAs (lncRNAs), which are more than 200 nucleotides in length, have been shown to play crucial regulatory roles in numerous biological processes [9, 10]. The mechanisms of action of lncRNAs are multifactorial and largely dependent on the specific intracellular localization of the molecule [11]. MicroRNAs (miRNAs) are a class of short noncoding RNAs (~22 nucleotides in length) [12, 13] that inhibit the expression of target genes by binding to the 3′ untranslated region (3′-UTR) of specific mRNA targets and hence degrade the mRNA or suppress translation [14]. In recent years, the “competitive endogenous RNA” (ceRNA) hypothesis has been proposed, and several studies have suggested the occurrence of interactions between lncRNAs and miRNAs [15–17], adding to the complexity of interactions between diverse RNA species. Despite rapidly rising interest in the expression and function of lncRNAs in cellular senescence [18–20], their potential implications in skin photoaging remain virtually unexplored.

In the previous study, we initially found that *RP11-670E13.6* was up-regulated in UVB-irradiated HDFs and delayed cellular senescence through the p16-pRB pathway [21]. In this study, we further investigated the functions and the regulatory mechanisms of *RP11-670E13.6* in HDFs. Our results provided important insights into the *RP11-670E13.6/miR-663a*/*CDK4* and *RP11-670E13.6/miR-663a*/ *CDK6* axis as ceRNA networks in UVB-induced cellular senescence. Moreover, we found that heterogeneous nuclear ribonucleoprotein H (hnRNPH) physically interacted with *RP11-670E13.6* and blocked its expression.

## RESULTS

### UVB up-regulated *RP11-670E13.6* in a ROS-independent manner, and knockdown of *RP11-670E13.6* promoted cellular senescence

**Figure 1:**
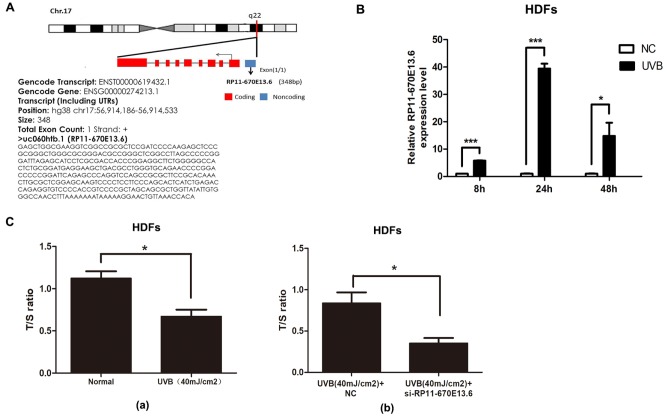
**UVB up-regulated *RP11-670E13.6* levels, and knockdown of *RP11-670E13.6* promoted cellular senescence.** (**A**) Schematic diagram of the localization of *RP11-670E13.6*. (**B**) Expression of *RP11-670E13.6* in the UVB irradiation and control groups, as determined by qRT-PCR. Data are shown as the means ± standard errors of the means based on at least three independent experiments. (**C**) (**a**) UVB irradiation decreased the mean length of telomeres in HDFs at 24 h post-irradiation. (**b**) Knockdown of *RP11-670E13.6* decreased the mean length of telomeres in HDFs at 24 h post-irradiation. Data are shown as the means ± standard errors of the means based on at least three independent experiments. *P* values were determined by Student’s *t*-tests. *P < 0.05; **P < 0.01; and ***P < 0.001.

*RP11-670E13.6* is a lncRNA consisting of one exon of 348 bp and located upstream of the *TRIM25* gene locus in chromosome 17 (Figure 1A). As shown in Figure 1B, *RP11-670E13.6* expression was significantly elevated in UVB-irradiated HDFs over time and the greatest increase was at 24 h after UVB irradiation.

In the previous study, we found that the ratio of senescent cells markedly increased following transfection with small-interfering RNA (siRNA) targeting *RP11-670E13.6* compared with that of the negative controls (NC) [21]. It has been postulated that telomere shortening played an important role in photoaging [22]. Senescence in primary HDFs can be triggered by telomere erosion [23]. In this study, relative quantitative real-time polymerase chain reaction analysis confirmed the β-galactosidase staining findings, showing that the mean telomere length decreased in *RP11-670E13.6* depleted HDFs at 24 h post-irradiation (Figure 1Cb). Moreover, the mean length of telomeres in UVB-irradiated HDFs decreased, suggesting that acute photodamage might contribute to early photoaging in human skin as a consequence of rapid telomere shortening (Figure 1Ca).

UV-induced ROS production is responsible for both clinical and biochemical manifestations of skin photoaging [24], and antioxidant enzymes, including catalase (CAT) and superoxide dismutase (SOD), are important for modulating ROS by scavenging free radicals in cells. To further investigate whether *RP11-670E13.6* expression was required for modulating ROS generation or vice versa, we pretreated cells with a ROS scavenger (N-acetyl-Lcysteine, [NAC], 10 mM) before detection of *RP11-670E13.6.* As anticipated, 40 mJ/cm^2^ UVB exposure significantly increased ROS generation, and NAC caused a reduction in UVB-induced ROS generation (Supplementary Figure 1A). However, NAC had no significant effect on UVB-induced up-regulation of *RP11-670E13.6* (Supplementary Figure 1B), neither generation of ROS nor SOD and CAT activity in UVB-irradiated HDFs were altered by *RP11-670E13.6* reduction (Supplementary Figure 1C–1E).

### Knockdown of *RP11-670E13.6* induced DNA damage

**Figure 2:**
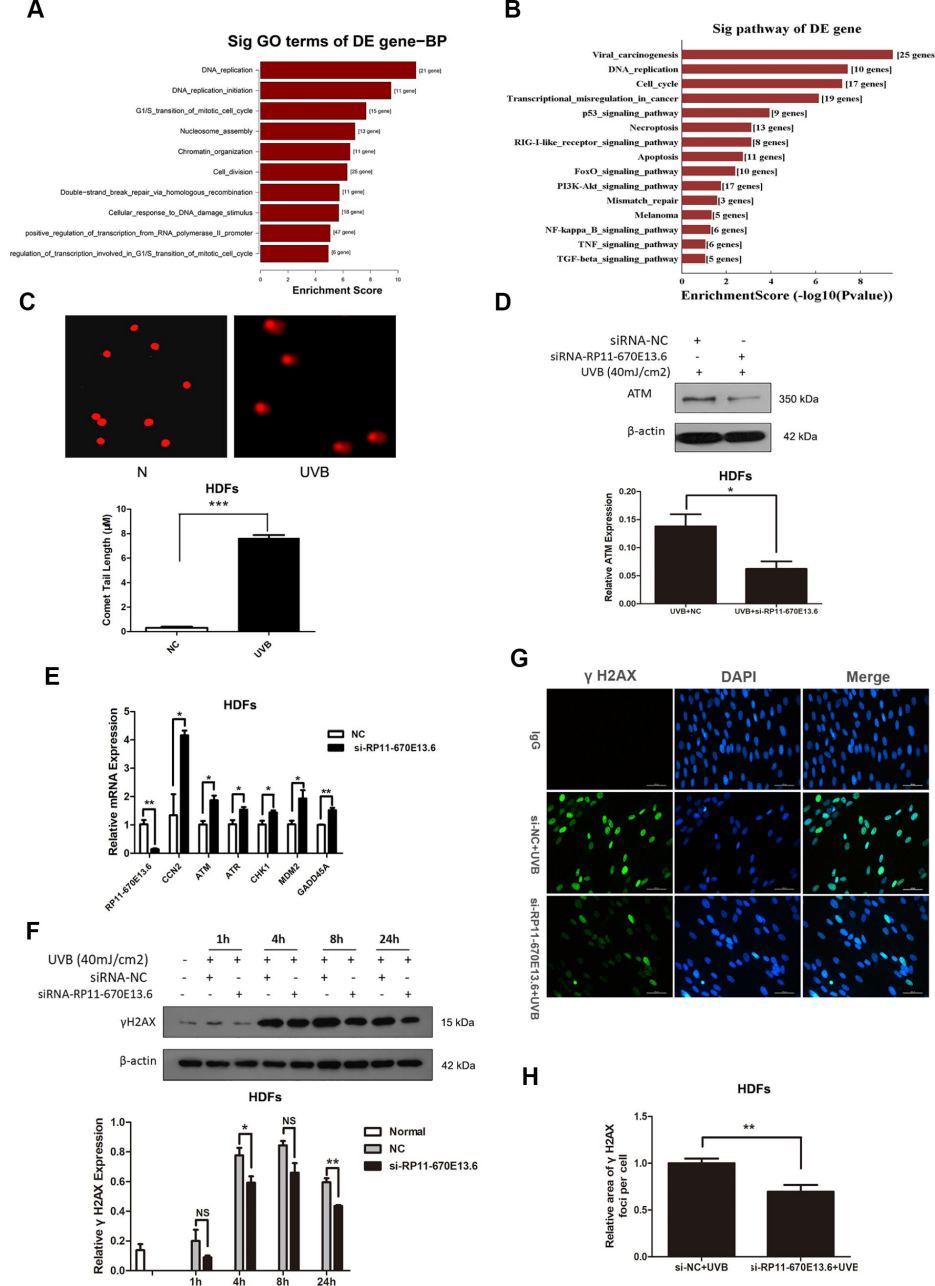
***RP11-670E13.6* promoted DNA damage repair.** (**A**) Top significant biological processes for genes whose transcript levels were increased in *RP11-670E13.6*-depleted HDFs. (**B**) Top significant Kyoto Encyclopedia of Genes and Genomes pathways for genes whose transcript levels were increased in *RP11-670E13.6*-depleted HDFs. (**C**) Comet tail length was quantified at 24 h after 40 mJ/cm^2^ UVB irradiation. Representative images are shown. Data are shown as the means ± standard errors of the means. (**D**) Representative image of western blotting results for the effects of *RP11-670E13.6* on the expression of ATM protein in HDFs. (**E**) Relative expression of the indicated DNA damage-associated genes was determined by qRT-PCR in *RP11-670E13.6*-depleted HDFs and negative controls. Data are shown as the means ± standard errors of the means based on at least three independent experiments. (**F**) HDFs were mock treated or transfected with siRNA against *RP11-670E13.6*. Two days after transfection, the cells were UVB (40mJ/cm^2^) irradiated and analyzed for H2AX phosphorylation at the indicated time points by western blot. (**G**) HDFs were mock treated or transfected with siRNA against *RP11-670E13.6*. Two days after transfection, the cells were UVB (40mJ/cm2) irradiated and analyzed for H2AX phosphorylation at 24h post-irradiation by immunofluorescent staining. (**H**) Quantification of γH2A.X foci expressed as mean relative area per cell. Twenty nuclei from the HDFs transfected with *RP11-670E13.6* siRNA and control siRNA were examined. *P* values were determined by Student’s *t*-tests. *P < 0.05; **P < 0.01; and ***P < 0.001.

To elucidate the molecular mechanisms through which *RP11-670E13.6* affected UVB-damaged HDFs, we performed expression profiling of HDFs transfected with *RP11-670E13.6* siRNA or siRNA NC using RNA-seq (Supplementary Figure 2A). Differentially expressed genes in *RP11-670E13.6* knockdown HDFs were significantly associated with specific gene ontology (GO) terms and Kyoto Encyclopedia of Genes and Genomes (KEGG) pathways. In *RP11-670E13.6*-delepted HDFs, significantly enriched GO terms included biological processes, such as DNA replication (*P* < 4.9E-12), G_1_/S transition of the mitotic cell cycle (*P* < 2.1E-08; Figure 2A), nucleosome assembly (*P* < 1.3E-07), chromatin organization (*P* < 3.1E-07), and double-strand breaks (DSBs) repair via homologous recombination (*P* < 1.9E-06). Molecular functions, such as protein binding (*P* < 2.9E-06), helicase activity (*P* < 2.7E-05), and DNA binding (*P* < 3.9E-05) were also affected (Supplementary Figure 2B). Moreover, significantly enriched KEGG pathways included viral carcinogenesis (*P* < 3.7E-10), DNA replication (*P* < 3.8E-08), cell cycle (*P* < 6.6E-08), and transcriptional misregulation in cancer (*P* < 7.4E-07; Figure 2B). These findings were consistent with our previous study that knockdown of *RP11-670E13.6* decreased HDFs proliferation and induced cell cycle arrest.

Because the mRNA expressions of many genes involving in DNA replication and DSBs repair were significantly altered by *RP11-670E13.6* depletion, we further examined whether *RP11-670E13.6* played a role in the DNA damage response (DDR) in UVB irradiated HDFs. Comet assays revealed an increase in the tail length of HDFs at 24 h after 40 mJ/cm^2^ UVB exposure (Figure 2C), suggesting that the UVB dose of 40 mJ/cm^2^ could induce DNA DSBs in HDFs. Moreover, our results showed that *RP11-670E13.6* depletion reduced the protein levels of ataxia telangiectasia mutated (ATM), which play a key role in UV damage signaling. (Figure 2D) [25, 26]. However, mRNA levels of *ATM,* in addition to many other genes involved in the DDR were significantly up-regulated by *RP11-670E13.6* depletion (Figure 2E). It is well known that DSBs formation at late time points after UV treatment activates ATM kinase activity, which then contributes to the increase of phosphorylation of Ser139 of histone H2A.X molecules (γH2A.X) [27]. Our results showed that the phosphorylation of H2A.X was also decreased by treatment with an siRNA targeting *RP11-670E13.6* in UVB-irradiated (40 J/m2) HDFs (Figure 2F). Immunofluorescence microscopic analyses showed that γH2A.X foci were also decreased in the *RP11-670E13.6* depleted HDFs than in controls (Figure 2G). The relative area of γH2A.X was significantly lesser in the *RP11-670E13.6*-depleted HDFs at 24 h after UVB irradiation than in control HDFs (Figure 2H).

### Cellular distribution of *RP11-670E13.6* in HDFs

**Figure 3:**
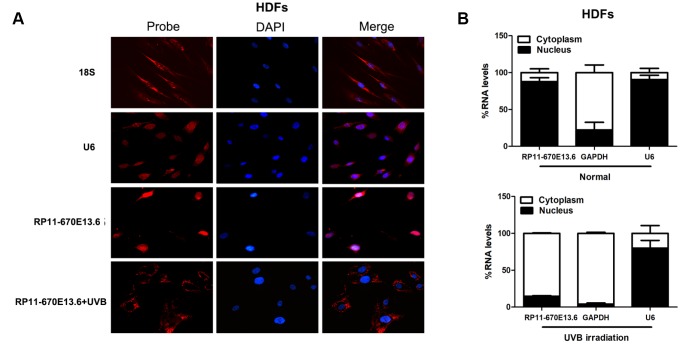
***RP11-670E13.6* cellular localization.** (**A**) FISH images showing localization of RP11-670E13.6 in HDFs treated with or without UVB irradiation for 24 h. (**B**) Percentage of nuclear and cytoplasmic RNA levels of *RP11-670E13.6*, *U6* and *GAPDH* measured by qRT-PCR after subcellular fractionation in HDFs irradiated or not irradiated with UVB for 24 h. Data are shown as the means ± standard errors of the means based on at least three independent experiments. *P* values were determined by Student’s *t*-tests. *P < 0.05; **P < 0.01; and ***P < 0.001. FISH, fluorescence in situ hybridization; 18S, probe for 18S rRNA; U6, probe for U6 snRNA.

To further study the underlying mechanisms through which *RP11-670E13.6* regulated cellular senescence, we examined the cellular distribution of *RP11-670E13.6* in HDFs under physiological and UVB-irradiated conditions. In control cells (physiological conditions), fluorescence in situ hybridization (FISH) revealed *RP11-670E13.6* in the nucleus, whereas it was detected in the cytoplasm after UVB irradiation (Figure 3A). By using cytoplasmic and nuclear RNA fractions from HDFs, we observed that *RP11-670E13.6* is expressed in relative abundance in the cytoplasm after UVB irradiation, which confirmed the results of FISH (Figure 3B).

As a newly described regulatory mechanism, a cytoplasmic lncRNA can act as a natural miRNA sponge, which interferes with miRNA pathways and reduces binding of endogenous miRNAs to target genes at the post-transcriptional level [28, 29]. Using an online bioinformatics website RNA22 version 2.0 (https://cm.jefferson.edu/), we identified a set of candidate miRNAs having putative binding sites with *RP11-670E13.6*. Incidentally, among them, we found several miRNAs also have putative binding sites with *CDK4*, *CDK6* and *CCND1.* As we found that knockdown of *RP11-670E13.6* decreased expression of Cdk4, Cdk6 and CyclinD1 [21], we speculated that *RP11-670E13.6* may affect Cdk4, Cdk6 and CyclinD1 expression via modulation of miRNAs in the cytoplasm of HDFs after UVB irradiation.

To test this hypothesis, several miRNA candidates that have putative binding sites with *CDK4*, *CDK6* and *CCND1* were selected to perform dual-luciferase reporter assays, and our data showed that *miR-663a* overexpression decreased the luciferase activity of the wild-type (WT) *RP11-670E13.6* reporter the most (Supplementary Figure 3A). Thus, we selected *miR-663a* to further investigate the association of *RP11-670E13.6* and *miR-663a* in UVB-induced cellular senescence.

### *MiR-663a* promoted cellular senescence by targeting *CDK4* and *CDK6*

**Figure 4:**
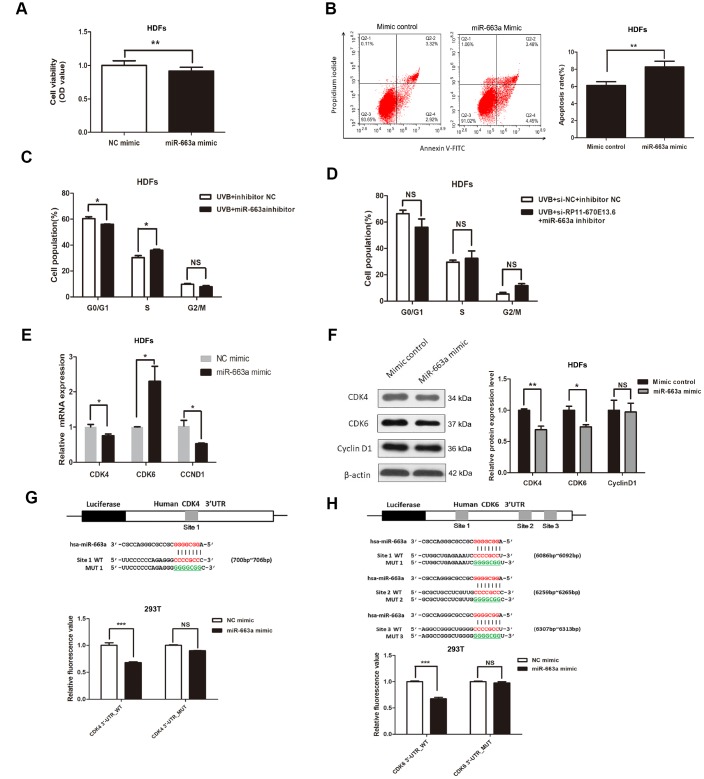
***miR-663a* promoted cellular senescence by targeting *CDK4* and *CDK6*.** (**A**) CCK-8 assays were used to detect the effect of *miR-663a* on HDF viability. Data are shown as the means ± standard errors of the means based on at least three independent experiments. (**B**) Flow cytometry depicted the percentages of apoptosis in HDFs transfected with miRNA mimics control and miR-663a mimics. (**C**) After miRNA inhibitor transfection for 48h, the cell cycle distribution of HDFs at 24 h post-UVB irradiation. (**D**) After cotransfection with siRNA and miRNA inhibitor for 48h, the cell cycle distribution of HDFs at 24 h post-UVB irradiation. (**E**) *miR-663a* negatively regulated the expression of *CDK4* and *CCND1*, but positively regulated *CDK6* at mRNA levels. (**F**) *miR-663a* negatively regulated the expression of Cdk4 and Cdk6 at protein levels, but had no effect on the expression of CyclinD1. (**G**) Putative binding site of *miR-663a* in the 3′-UTR of *CDK4* and the sites of target mutagenesis are indicated. Luciferase activity in HDFs, demonstrating the effects of *miR-663a* on the expression of its target gene *CDK4*. (**H**) Putative binding site of *miR-663a* in the 3′-UTR of *CDK6* and the sites of target mutagenesis are indicated. Luciferase activity in HDFs, demonstrating the effects of *miR-663a* on the expression of its target gene *CDK6*. Data are shown as the means ± standard errors of the means based on at least three independent experiments. *P* values were determined by Student’s *t*-tests. *P < 0.05; **P < 0.01; and ***P < 0.001.

To investigate the biological functions of *miR-663a* in cellular senescence upon UVB exposure, we explored the potential effects of *miR-663a* on proliferation, apoptosis and cell cycle progression. As shown in Figure 4A and Figure 4B, *miR-663a* mimic inhibited the proliferation and stimulated apoptosis of HDFs. Cell cycle analysis showed that treatment of *miR-663a* inhibitor drove progression beyond the G1/S transition in UVB-irradiated HDFs (Figure 4C). To test whether *RP11-670E13.6* depletion caused defects in the G1-to-S transition by interacting with *miR-663a*, we cotransfection with *RP11-670E13.6* siRNA and miR-663a inhibitor in HDFs, and failed to observe G1/S arrest in *RP11-670E13.6* depleted HDFs (Figure 4D).

Next, we verified the predicted target regulation relationship between *CDK4/CDK6/CCND1* and *miR-663a* by quantitative reverse transcription polymerase chain reaction (qRT-PCR) and western blotting in HDFs. Consistent with the fluorescence-activated cell sorting data, the expression of G1/S phase checkpoint proteins such as Cdk4 and Cdk6 were down-regulated in cells with *miR-663a* overexpression (Figure 4F). Moreover, *miR-663a* inhibited the expression of *CDK4* mRNA, whereas increased the *CDK6* mRNA levels (Figure 4E). In addition, our results showed that *miR-663a* had no effect on CyclinD1 expression, though it decreased *CCND1* mRNA expression (Figure 4F).

To further investigate whether the suppression of Cdk4 and Cdk6 occurred via the potential interactions at putative *miR-663a*-binding sites, we generated different mutants (MUTs) and found out that *miR-663a* overexpression significantly decreased luciferase activities of the *CDK4* and *CDK6* WT reporters, but did not affect that of the mutant reporters (Figure 4G, Figure 4H), indicating that *miR-663a* directly bound to the 3′-UTR of *CDK4* and *CDK6* mRNA. Additionally, *miR-663a* overexpression significantly decreased luciferase activities both of the *CCND1* WT and MUT reporters, indicating that *CCND1* was not a direct target of *miR-663a* (Supplementary Figure 3B).

### *RP11-670E13.6* acted as sponge for *miR-663a*

**Figure 5:**
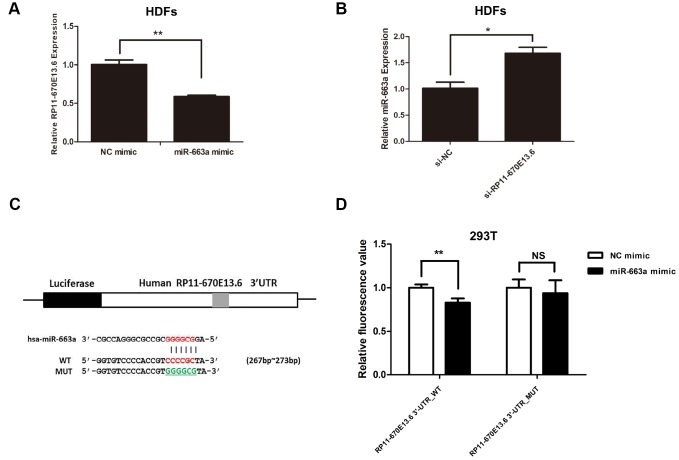
**Reciprocal repression of *RP11-670E13.6* and *miR-663a*.** (**A**) *miR-663a* negatively regulated the expression of its target gene *RP11-670E13.6*. (**B**) *RP11-670E13.6* negatively regulated the expression of *miR-663a*. (**C**) Putative binding site of *miR-663a* in *RP11-670E13.6* and the site of target mutagenesis are indicated. (**D**) Luciferase activity in HDFs, demonstrating the effects of *miR-663a* on the expression of its target gene *RP11-670E13.6*. Data are shown as the means ± standard errors of the means based on at least three independent experiments. *P* values were determined by Student’s *t*-tests. *P < 0.05; **P < 0.01; and ***P < 0.001.

To further study the relationship between *RP11-670E13.6* and *miR-663a,* we found that *miR-663a* overexpression inhibited *RP11-670E13.6* expression by approximately 42% (Figure 5A), whereas *RP11-670E13.6* knockdown increased *miR-663a* expression (Figure 5B). In our next experiment, luciferase reporter constructs were generated (Figure 5C), and dual-luciferase assays showed a significant decrease in luciferase activities after cotransfection with *miR-663a* mimic and the WT *RP11-670E13.6* expression vector, but not a MUT *RP11-670E13.6* expression vector (Figure 5D), indicating that *miR-663a* bound directly to *RP11-670E13.6* and that the binding sites were vital for reciprocal repression of *RP11-670E13.6* and *miR-663a*. Thus, these data indicated that *RP11-670E13.6* acted as an endogenous “sponge” by binding *miR-663a*, which abolished the repressive effects of *miR-663a* on the Cdk4 and Cdk6 expression.

### hnRNPH directly bound to and suppressed *RP11-670E13.6* expression

**Figure 6:**
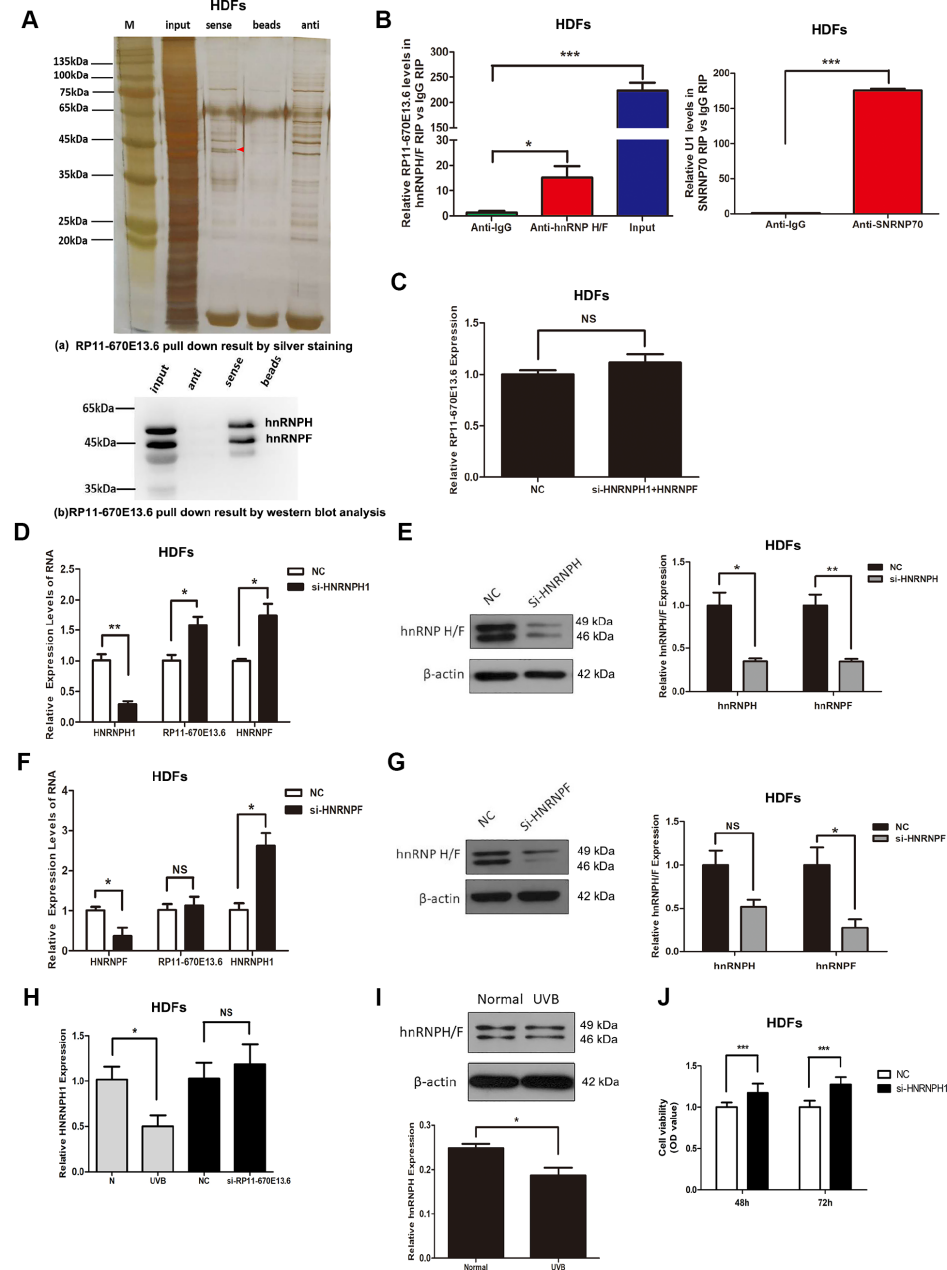
**hnRNPH directly bound to *RP11-670E13.6* and repressed its expression.** (**A**) Pull down results of RP11-670E13.6 by silver staining (**a**) and western blot analysis (**b**) demonstrated the possible interactions between *RP11-670E13.6* and hnRNPF/H. (**B**) RIP assays demonstrating the enrichment of hnRNPF/H on *RP11-670E13.6* transcripts relative to IgG in HDFs. (**C**) Knockdown of both hnRNPH and hnRNPF had no effect on the expression of *RP11-670E13.6*. (**D**) Effects of *HNRNPH1* siRNA on the expression of *RP11-670E13.6* and hnRNPF. (**E**) Effects of *HNRNPF* siRNA on the expression of *RP11-670E13.6* and hnRNPH. (**F**) The mRNA expression levels of *HNRNPH1*. (**G**) HnRNPH/F expression levels of HDFs treated with *RP11-670E13.6* siRNA and UVB irradiation. (**H**) The mRNA expression levels of *HNRNPH1*. (**I**) hnRNP H/F expression levels of HDFs treated with UVB irradiation(40mJ/cm^2^). (**J**) CCK-8 assays were used to detect the effects of *HNRNPH1* on HDFs viability. Data are shown as the means ± standard errors of the means based on at least three independent experiments. *P* values were determined by Student’s *t*-tests. *P < 0.05; **P < 0.01; and ***P < 0.001.

RNA-binding proteins (RBPs) that function as alternative splicing regulators bind to pre-mRNA *cis*-acting elements and can promote or repress spliceosome formation and regulate alternative splice site usage in the mature transcript [30]. To identify RBPs associated with *RP11-670E13.6* production, we used affinity pulldown analysis, mass spectrometry, and immunoblotting and revealed a direct interaction between *RP11-670E13.6* and hnRNPF/H (Figure 6A), which was further confirmed by RNA immunoprecipitation (RIP) assays (Figure 6B). Moreover, silencing of *HNRNPH* up-regulated *RP11-670E13.6* (Figure 6D), whereas *HNRNPF* had no effect on its expression (Figure 6C, 6F), suggesting *RP11-670E13.6* is a target of hnRNPH but not hnRNPF. Furthermore, we found that silencing of *HNRNPH* increased *HNRNPF* mRNA but decreased hnRNPF protein (Figure 6D, 6E), and vice versa (Figure 6F, 6G). As shown in Figure 6H and Figure 6I, UVB irradiation reduced hnRNPH expression at both the mRNA and protein levels, however, knockdown of *RP11-670E13.6* did not affect hnRNPH, suggesting *RP11-670E13.6* may be a downstream target of hnRNPH. Additionally, we found that silencing of *HNRNPH* promoted HDFs proliferation (Figure 6J), consistent with the biological functions of increased *RP11-670E13.6*.

## DISCUSSION

**Figure 7:**
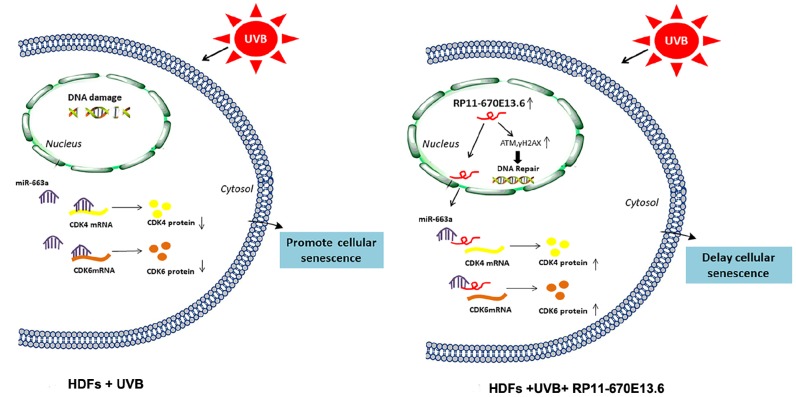
**Schematic diagram of the hypothesis that lncRNA *RP11-670E13.6* delayed UVB induced cellular senescence by facilitating DNA damage repair and competing for *miR-663a* to up-regulate Cdk4 and Cdk6 expression in HDFs.**

In the current study, we demonstrated that the lncRNA *RP11-670E13.6*, interacted with hnRNPH, delayed cellular senescence by facilitating DNA damage repair and increasing Cdk4 and Cdk6 levels in UVB damaged HDFs (Figure 7). Briefly, hnRNPH suppressed expression of *RP11-670E13.6* under physiological conditions. When UVB irradiation down-regulated hnRNPH, *RP11-670E13.6* expression was significantly increased in a ROS-independent manner and facilitating DNA damage repair by increasing the kinase activity of ATM and the phosphorylation of histone H2A.X molecules. Moreover, upon UVB irradiation, *RP11-670E13.6* translocated from the nucleus to the cytoplasm. In the cytoplasm, *RP11-670E13.6* functioned as an endogenous “sponge” by binding to *miR-663a*, abolishing the repressive activities of *miR-663a* on Cdk4 and Cdk6, and thereby delaying UVB-induced cellular senescence.

Telomere length is a molecular marker of cell aging, and genomic instability due to telomere shortening has been linked to aging-related diseases [31]. Recent studies have suggested that intrinsic aging and photoaging share a common pathway involving telomere-generated signaling that is responsible for most clinical manifestations of skin [32]. In this study, we found that knocked down *RP11-670E13.6* decreased mean telomere length in UVB irradiated HDFs, indicating that *RP11-670E13.6* delayed UVB-induced cellular senescence. It is well known that cells undergo senescence in response to severely damaged DNA [33, 34]. The DNA damage repair is characterized by the activation of ATM and ATR [35], which are recruited to the site of damage and lead to phosphorylation of histone H2A.X. Phosphorylated H2AX can be visualized as foci by immunofluorescence using phospho-specific antibodies [36]. H2AX foci colocalize with foci of other proteins, including NBS1, 53BP1, MDC1, and BRCA1 [36–38]. Although the initial recruitment of these proteins appears to be γ-H2AX independent, their retention as foci at longer times post-irradiation does not occur in cells lacking H2AX, leading to the suggestion that γ-H2AX plays a critical role in the retention of repair factors at the sites of DSBs [39, 40]. One study examining ATM knockout cell lines concluded that IR-induced γ-H2AX foci formation is ATM dependent [41]. In our study, *RP11-670E13.6* depletion inhibited the kinase activity of ATM, which decreased the phosphorylation of H2A.X, leading to the DNA damage in UVB-irradiated HDFs not been repaired, and then inducing cellular senescence. Taken together, our results suggest that *RP11-670E13.6* may promote DNA damage repair by increasing ATM and γH2A.X expression in UVB irradiated HDFs, and thereby delaying cellular senescence.

Using bioinformatics analysis, we found that *miR-663a* formed complementary base pairing with *CDK4, CDK6* and *RP11-670E13.6*, and luciferase reporter assays confirmed that these molecules were direct targets of *miR-663a*. It has been described that *miR-663a* inhibited cell proliferation and invasion by targeting JunD in human non-small cell lung cells and *miR-663* may regulate the proliferation of fibroblasts in hypertrophic scar [42, 43]. In this study, cell cycle analysis and cell proliferation activity analysis showed that *miR-663a* inhibited cell growth and induced cell cycle arrest. Moreover, our experiments revealed that overexpression of *miR-663a* repressed Cdk4 and Cdk6 by targeting the 3′-UTR of *CDK4* and *CDK6*. We have revealed that *RP11-670E13.6* depletion may cause defects in the G1-to-S transition previously. Here, we showed that *RP11-670E13.6* depletion could not inhibited G1-S transition after transfection with*miR-663a* inhibitor in HDFs, suggesting that *RP11-670E13.6* may up-regulate Cdk4 and Cdk6 expression by interacted with *miR-663a*. Furthermore, we have observed a negative regulation between *RP11-670E13.6* and *miR-663a*, providing evidence to the reciprocal repression of *RP11-670E13.6* and *miR-663a*. Here, we only discussed the function that *miR-663a* was targeted by *RP11-670E13.6*, and *miR-663a* targeted *RP11-670E13.6* was remain to be explored.

It is known that miRNAs negatively regulate gene expression at the post-transcriptional level, mainly via binding to the 3′- UTR of the target gene. The binding of the miRNA with target mRNA may lead to blockage of protein translation as well as reduced mRNA stability, and the latter seems to be the predominant mechanism in miRNA-dependent gene repression [44]. We showed that *miR-663a* overexpression decreased *CDK4* mRNA level and increased *CDK6* level, indicating that *miR-663a* may inhibit the expression of Cdk4 and Cdk6 by degrading the *CDK4* mRNA and suppressing Cdk6 protein translation. The activities and functions of lncRNAs are thought to depend on their subcellular distribution [45]. Herein, we observed that *RP11-670E13.6* was localized in the nucleus under physiological condition, but almost exclusively in the cytoplasm following UVB irradiation, therefore, its function as a ceRNA could be attributed to its cytoplasmic localization. However, its roles in the nuclear compartment were not investigated herein. Nuclear biogenesis of *RP11-670E13.6* may explain its localization in the nucleus, although we speculate that nuclear processes, such as transcription or epigenetic regulation, could be involved, similar to other previously described lncRNAs [46–48].

In vitro, cellular senescence happens in 2 steps: cell cycle arrest followed, or sometimes preceded, by gerogenic conversion (geroconversion). Geroconversion is a form of growth, a futile growth during cell cycle arrest. It converts reversible arrest to irreversible senescence, which is driven in part by the growth-promoting mTOR pathway [49–51]. It is known that telomere erosion promotes DNA damage responsive signals, thereby causing irreversible cell-cycle arrest [52]. In our study, knocked down *RP11-670E13.6* decreased mean telomere length and induced serious DNA damage in UVB-irradiated HDFs, suggesting *RP11-670E13.6* depletion induce an irreversible state of cell-cycle. Moreover, in UV-treated cells, mTOR remained fully active [53]. Thus, we considered that knocked down *RP11-670E13.6* promote cellular senescence partly by inducing cell cycle arrest in UVB-irradiated HDFs.

An important aspect of our findings concerns hnRNPH. Our results showed that hnRNPH directly bound to and suppressed *RP11-670E13.6* expression. Although hnRNPH-dependent regulation of splicing was linked to the closely related protein hnRNPF [54], we found that silencing of *HNRNPF* had no effect on *RP11-670E13.6* expression. Moreover, our data showed that hnRNPH protein were downregulated in UVB-irradiated HDFs compared with that in non-irradiated cells, and ectopic low expression of *HNRNPH* increased the relative levels of *RP11-670E13.6* and promoted HDFs proliferation, consistent with our previous report demonstrating that knockdown of *RP11-670E13.6* inhibited cell proliferation [21]. Thus, we identified hnRNPH as a factor that repressed HDFs proliferation at least in part by inhibiting the production of *RP11-670E13.6*, although other RNA targets of hnRNPH almost certainly also contributed to preventing cell proliferation.

In summary, we propose a mechanism through which lncRNA *RP11-670E13.6* delayed cellular senescence by facilitating DNA damage repair and competing for *miR-663a* to up-regulate Cdk4 and Cdk6 expression in UVB damaged HDFs. Moreover, we presented strong evidence that hnRNPH physically interacted with *RP11-670E13.6* and blocked its expression.

## MATERIALS AND METHODS

### Cell culture and UV irradiation

293T cells were purchased from the Cell Bank of the Chinese Academy of Science (Shanghai, China). Primary HDFs were cultured from normal human foreskin specimens obtained from circumcision surgery in our clinic and cultured in Dulbecco’s modified Eagle’s medium (HyClone, Logan, UT, USA) supplemented with 10% fetal bovine serum (Gibco BRL, Grand Island, NY, USA) and 1% penicillin/streptomycin (HyClone) at 37°C in the presence of 5% CO_2_. HDFs were used from passages 3 to 8 in all experiments. Each experiment was repeated in HDFs at least from three different individuals. UVB irradiations were performed using a Waldmann UV 208T lamp (Herbert Waldmann GmbH & Co, Villingen-Schwenningen, Germany) with a peak emission wavelength of 313 nm as previously reported [21].

### RNA- seq

Sequencing was performed at Shanghai KangChen Bio-tech, and RNA-seq data were aligned to the reference genome (human assembly GRCh37/hg19) using Tophat2 (http://ccb.jhu.edu/software/tophat). HTSeq (http://www-huber.embl.de/HTSeq) was then applied on the aligned data set to determine differentially expressed genes with a “significant” status. GO and KEGG analyses of differentially expressed genes were performed using DAVID (https://david.ncifcrf.gov/).

### Cell treatments and other techniques

Detailed protocols describing cell treatments and other experimental techniques are presented in the Supplementary Materials.

### Statistical analysis

All data are expressed as means ± standard errors of at least three independent experiments. All statistical analyses were carried out using GraphPad Prism 5 Software. Differences between groups were analyzed using Student’s *t*-tests. In cases of multiple-group testing, one-way analysis of variance was conducted. Differences with *P* values of less than 0.05 were considered statistically significant.

## Supplementary Material

Supplementary Materials

Supplementary Figures

Supplementary Tables

## References

[1] Gilchrest BA. Photoaging. J Invest Dermatol. 2013; 133:E2–6. 10.1038/skinbio.2013.176 23820721

[2] Fisher GJ, Wang ZQ, Datta SC, Varani J, Kang S, Voorhees JJ. Pathophysiology of premature skin aging induced by ultraviolet light. N Engl J Med. 1997; 337:1419–28. 10.1056/NEJM199711133372003 9358139

[3] Warren R, Gartstein V, Kligman AM, Montagna W, Allendorf RA, Ridder GM. Age, sunlight, and facial skin: a histologic and quantitative study. J Am Acad Dermatol. 1991; 25:751–60. 10.1016/S0190-9622(08)80964-4 1802896

[4] Childs BG, Gluscevic M, Baker DJ, Laberge RM, Marquess D, Dananberg J, van Deursen JM. Senescent cells: an emerging target for diseases of ageing. Nat Rev Drug Discov. 2017; 16:718–35. 10.1038/nrd.2017.116 28729727 PMC5942225

[5] Ichihashi M, Ueda M, Budiyanto A, Bito T, Oka M, Fukunaga M, Tsuru K, Horikawa T. UV-induced skin damage. Toxicology. 2003; 189:21–39. 10.1016/S0300-483X(03)00150-1 12821280

[6] Trautinger F. Mechanisms of photodamage of the skin and its functional consequences for skin ageing. Clin Exp Dermatol. 2001; 26:573–77. 10.1046/j.1365-2230.2001.00893.x 11696060

[7] Sedelnikova OA, Horikawa I, Zimonjic DB, Popescu NC, Bonner WM, Barrett JC. Senescing human cells and ageing mice accumulate DNA lesions with unrepairable double-strand breaks. Nat Cell Biol. 2004; 6:168–70. 10.1038/ncb1095 14755273

[8] Haferkamp S, Tran SL, Becker TM, Scurr LL, Kefford RF, Rizos H. The relative contributions of the p53 and pRb pathways in oncogene-induced melanocyte senescence. Aging (Albany NY). 2009; 1:542–56. 10.18632/aging.100051 20157537 PMC2806033

[9] Lalevée S, Feil R. Long noncoding RNAs in human disease: emerging mechanisms and therapeutic strategies. Epigenomics. 2015; 7:877–79. 10.2217/epi.15.55 26418705

[10] Ponting CP, Oliver PL, Reik W. Evolution and functions of long noncoding RNAs. Cell. 2009; 136:629–41. 10.1016/j.cell.2009.02.006 19239885

[11] Kopp F, Mendell JT. Functional Classification and Experimental Dissection of Long Noncoding RNAs. Cell. 2018; 172:393–407. 10.1016/j.cell.2018.01.011 29373828 PMC5978744

[12] Bartel DP. MicroRNAs: target recognition and regulatory functions. Cell. 2009; 136:215–33. 10.1016/j.cell.2009.01.002 19167326 PMC3794896

[13] Thomas M, Lieberman J, Lal A. Desperately seeking microRNA targets. Nat Struct Mol Biol. 2010; 17:1169–74. 10.1038/nsmb.1921 20924405

[14] Zhou BR, Guo XF, Zhang JA, Xu Y, Li W, Wu D, Yin ZQ, Permatasari F, Luo D. Elevated miR-34c-5p mediates dermal fibroblast senescence by ultraviolet irradiation. Int J Biol Sci. 2013; 9:743–52. 10.7150/ijbs.5345 23983607 PMC3753410

[15] Cesana M, Cacchiarelli D, Legnini I, Santini T, Sthandier O, Chinappi M, Tramontano A, Bozzoni I. A long noncoding RNA controls muscle differentiation by functioning as a competing endogenous RNA. Cell. 2011; 147:358–69. 10.1016/j.cell.2011.09.028 22000014 PMC3234495

[16] Salmena L, Poliseno L, Tay Y, Kats L, Pandolfi PP. A ceRNA hypothesis: the Rosetta Stone of a hidden RNA language? Cell. 2011; 146:353–58. 10.1016/j.cell.2011.07.014 21802130 PMC3235919

[17] Tay Y, Rinn J, Pandolfi PP. The multilayered complexity of ceRNA crosstalk and competition. Nature. 2014; 505:344–52. 10.1038/nature12986 24429633 PMC4113481

[18] Kour S, Rath PC. Long noncoding RNAs in aging and age-related diseases. Ageing Res Rev. 2016; 26:1–21. 10.1016/j.arr.2015.12.001 26655093

[19] Abdelmohsen K, Panda A, Kang MJ, Xu J, Selimyan R, Yoon JH, Martindale JL, De S, Wood WH 3rd, Becker KG, Gorospe M. Senescence-associated lncRNAs: senescence-associated long noncoding RNAs. Aging Cell. 2013; 12:890–900. 10.1111/acel.12115 23758631 PMC3773026

[20] Ghanam AR, Xu Q, Ke S, Azhar M, Cheng Q, Song X. Shining the Light on Senescence Associated LncRNAs. Aging Dis. 2017; 8:149–61. 10.14336/AD.2016.0810 28400982 PMC5362175

[21] Li M, Li L, Zhang X, Yan Y, Wang B. LncRNA RP11-670E13.6 Regulates Cell Cycle Progression in UVB Damaged Human Dermal Fibroblasts. Photochem Photobiol. 2018; 94:589–97. 10.1111/php.12858 29143326

[22] Kosmadaki MG, Gilchrest BA. The role of telomeres in skin aging/photoaging. Micron. 2004; 35:155–59. 10.1016/j.micron.2003.11.002 15036269

[23] Bodnar AG, Ouellette M, Frolkis M, Holt SE, Chiu CP, Morin GB, Harley CB, Shay JW, Lichtsteiner S, Wright WE. Extension of life-span by introduction of telomerase into normal human cells. Science. 1998; 279:349–52. 10.1126/science.279.5349.349 9454332

[24] Wlaschek M, Tantcheva-Poór I, Naderi L, Ma W, Schneider LA, Razi-Wolf Z, Schüller J, Scharffetter-Kochanek K. Solar UV irradiation and dermal photoaging. J Photochem Photobiol B. 2001; 63:41–51. 10.1016/S1011-1344(01)00201-9 11684450

[25] Liu W, Otkur W, Zhang Y, Li Q, Ye Y, Zang L, He H, Hayashi T, Tashiro S, Onodera S, Ikejima T. Silibinin protects murine fibroblast L929 cells from UVB-induced apoptosis through the simultaneous inhibition of ATM-p53 pathway and autophagy. FEBS J. 2013; 280:4572–84. 10.1111/febs.12426 23829351

[26] Andrade-Lima LC, Andrade LN, Menck CF. ATR suppresses apoptosis after UVB irradiation by controlling both translesion synthesis and alternative tolerance pathways. J Cell Sci. 2015; 128:150–59. 10.1242/jcs.161596 25380827

[27] Yajima H, Lee KJ, Zhang S, Kobayashi J, Chen BP. DNA double-strand break formation upon UV-induced replication stress activates ATM and DNA-PKcs kinases. J Mol Biol. 2009; 385:800–10. 10.1016/j.jmb.2008.11.036 19071136 PMC2662442

[28] Quinn JJ, Chang HY. Unique features of long non-coding RNA biogenesis and function. Nat Rev Genet. 2016; 17:47–62. 10.1038/nrg.2015.10 26666209

[29] Cao C, Sun J, Zhang D, Guo X, Xie L, Li X, Wu D, Liu L. The long intergenic noncoding RNA UFC1, a target of MicroRNA 34a, interacts with the mRNA stabilizing protein HuR to increase levels of β-catenin in HCC cells. Gastroenterology. 2015; 148:415–26.e18. 10.1053/j.gastro.2014.10.012 25449213

[30] Blencowe BJ. Alternative splicing: new insights from global analyses. Cell. 2006; 126:37–47. 10.1016/j.cell.2006.06.023 16839875

[31] Han J, Qureshi AA, Prescott J, Guo Q, Ye L, Hunter DJ, De Vivo I. A prospective study of telomere length and the risk of skin cancer. J Invest Dermatol. 2009; 129:415–21. 10.1038/jid.2008.238 18668136 PMC2632304

[32] Yaar M, Gilchrest BA. Photoageing: mechanism, prevention and therapy. Br J Dermatol. 2007; 157:874–87. 10.1111/j.1365-2133.2007.08108.x 17711532

[33] López-Otín C, Blasco MA, Partridge L, Serrano M, Kroemer G. The hallmarks of aging. Cell. 2013; 153:1194–1217. 10.1016/j.cell.2013.05.039 23746838 PMC3836174

[34] Nakamura AJ, Chiang YJ, Hathcock KS, Horikawa I, Sedelnikova OA, Hodes RJ, Bonner WM. Both telomeric and non-telomeric DNA damage are determinants of mammalian cellular senescence. Epigenetics Chromatin. 2008; 1:6. 10.1186/1756-8935-1-6 19014415 PMC2584625

[35] Rouse J, Jackson SP. Interfaces between the detection, signaling, and repair of DNA damage. Science. 2002; 297:547–51. 10.1126/science.1074740 12142523

[36] Paull TT, Rogakou EP, Yamazaki V, Kirchgessner CU, Gellert M, Bonner WM. A critical role for histone H2AX in recruitment of repair factors to nuclear foci after DNA damage. Curr Biol. 2000; 10:886–95. 10.1016/S0960-9822(00)00610-2 10959836

[37] Wang B, Matsuoka S, Carpenter PB, Elledge SJ. 53BP1, a mediator of the DNA damage checkpoint. Science. 2002; 298:1435–38. 10.1126/science.1076182 12364621

[38] Stewart GS, Wang B, Bignell CR, Taylor AM, Elledge SJ. MDC1 is a mediator of the mammalian DNA damage checkpoint. Nature. 2003; 421:961–66. 10.1038/nature01446 12607005

[39] Celeste A, Petersen S, Romanienko PJ, Fernandez-Capetillo O, Chen HT, Sedelnikova OA, Reina-San-Martin B, Coppola V, Meffre E, Difilippantonio MJ, Redon C, Pilch DR, Olaru A, et al. Genomic instability in mice lacking histone H2AX. Science. 2002; 296:922–27. 10.1126/science.1069398 11934988 PMC4721576

[40] Celeste A, Fernandez-Capetillo O, Kruhlak MJ, Pilch DR, Staudt DW, Lee A, Bonner RF, Bonner WM, Nussenzweig A. Histone H2AX phosphorylation is dispensable for the initial recognition of DNA breaks. Nat Cell Biol. 2003; 5:675–79. 10.1038/ncb1004 12792649

[41] Burma S, Chen BP, Murphy M, Kurimasa A, Chen DJ. ATM phosphorylates histone H2AX in response to DNA double-strand breaks. J Biol Chem. 2001; 276:42462–67. 10.1074/jbc.C100466200 11571274

[42] Zhang Y, Xu X, Zhang M, Wang X, Bai X, Li H, Kan L, Zhou Y, Niu H, He P. MicroRNA-663a is downregulated in non-small cell lung cancer and inhibits proliferation and invasion by targeting JunD. BMC Cancer. 2016; 16:315. 10.1186/s12885-016-2350-x 27184257 PMC4869303

[43] Chen Q, Zhao T, Xie X, Yu D, Wu L, Yu W, Sun W. MicroRNA-663 regulates the proliferation of fibroblasts in hypertrophic scars via transforming growth factor-β1. Exp Ther Med. 2018; 16:1311–17. 10.3892/etm.2018.6350 30116380 PMC6090240

[44] Baer C, Claus R, Plass C. Genome-wide epigenetic regulation of miRNAs in cancer. Cancer Res. 2013; 73:473–77. 10.1158/0008-5472.CAN-12-3731 23316035

[45] Mercer TR, Mattick JS. Structure and function of long noncoding RNAs in epigenetic regulation. Nat Struct Mol Biol. 2013; 20:300–07. 10.1038/nsmb.2480 23463315

[46] Engreitz JM, Haines JE, Perez EM, Munson G, Chen J, Kane M, McDonel PE, Guttman M, Lander ES. Local regulation of gene expression by lncRNA promoters, transcription and splicing. Nature. 2016; 539:452–55. 10.1038/nature20149 27783602 PMC6853796

[47] Xing Z, Lin A, Li C, Liang K, Wang S, Liu Y, Park PK, Qin L, Wei Y, Hawke DH, Hung MC, Lin C, Yang L. lncRNA directs cooperative epigenetic regulation downstream of chemokine signals. Cell. 2014; 159:1110–25. 10.1016/j.cell.2014.10.013 25416949 PMC4266991

[48] West JA, Davis CP, Sunwoo H, Simon MD, Sadreyev RI, Wang PI, Tolstorukov MY, Kingston RE. The long noncoding RNAs NEAT1 and MALAT1 bind active chromatin sites. Mol Cell. 2014; 55:791–802. 10.1016/j.molcel.2014.07.012 25155612 PMC4428586

[49] Blagosklonny MV. Cell cycle arrest is not yet senescence, which is not just cell cycle arrest: terminology for TOR-driven aging. Aging (Albany NY). 2012; 4:159–65. 10.18632/aging.100443 22394614 PMC3348476

[50] Blagosklonny MV. Geroconversion: irreversible step to cellular senescence. Cell Cycle. 2014; 13:3628–35. 10.4161/15384101.2014.985507 25483060 PMC4614001

[51] Demidenko ZN, Blagosklonny MV. Growth stimulation leads to cellular senescence when the cell cycle is blocked. Cell Cycle. 2008; 7:3355–61. 10.4161/cc.7.21.6919 18948731

[52] Bernadotte A, Mikhelson VM, Spivak IM. Markers of cellular senescence. Telomere shortening as a marker of cellular senescence. Aging (Albany NY). 2016; 8:3–11. 10.18632/aging.100871 26805432 PMC4761709

[53] Leontieva OV, Blagosklonny MV. While reinforcing cell cycle arrest, rapamycin and Torins suppress senescence in UVA-irradiated fibroblasts. Oncotarget. 2017; 8:109848–56. 10.18632/oncotarget.17827 29312653 PMC5752566

[54] Wang E, Aslanzadeh V, Papa F, Zhu H, de la Grange P, Cambi F. Global profiling of alternative splicing events and gene expression regulated by hnRNPH/F. PLoS One. 2012; 7:e51266. 10.1371/journal.pone.0051266 23284676 PMC3524136

[55] Cawthon RM. Telomere measurement by quantitative PCR. Nucleic Acids Res. 2002; 30:e47. 10.1093/nar/30.10.e47 12000852 PMC115301

[56] Olive PL, Banáth JP. The comet assay: a method to measure DNA damage in individual cells. Nat Protoc. 2006; 1:23–29. 10.1038/nprot.2006.5 17406208

