# Supplementary material for: LncRNA RP11-670E13.6, interacted with hnRNPH, delays cellular senescence by sponging microRNA-663a in UVB damaged dermal fibroblasts
Source: Aging (Albany NY). 2019 Aug 23;11(16):5992–6013. doi: 10.18632/aging.102159 (PMC6738423; doi:10.18632/aging.102159)
Supplement: Supplementary Materials [file aging-11-102159-s001.pdf]

SUPPLEMENTARY MATERIALS

RNA isolation and qRT-PCR analysis

Total RNA from HDFs was extracted using TRIzol Reagent (Invitrogen, Carlsbad, CA, USA) and then reverse transcribed to cDNA using a Revert Aid First Strand cDNA Synthesis Kit (Thermo Fisher Scientific). Real-time qPCR analysis was performed using a SYBR Fast qRT-PCR Master Mix Kit (Kapa Biosystems, Wilmington, MA, USA) and a Light Cycler 480 system (Roche, Basel, Switzerland) according to the manufacturer's instructions. For each sample and index, the samples were studied in triplicate, with *GAPDH* mRNA expression measured as an internal reference. The primer sequences used in the real-time PCR are listed in Supplementary Table 1. miRNA sequence-specific RT-qPCR for *miR-663a* and the endogenous control *U6* were performed using a Bulge-Loop miRNA qRT-PCR Starter Kit (RiboBio, Guangzhou, China) and Bulge-Loop miRNA qRT-PCR Primer (RiboBio). Fold changes were calculated using the relative quantification ($2^{-\Delta\Delta Ct}$) method.

Telomere length analysis

To measure telomere length, total DNA was extracted using a Genomic DNA Extraction Kit (Tiangen Biotech, Beijing, China) according to the manufacturer's manual. Genomic DNA was quantified using a UV-Vis spectrophotometer (Smart Spectro 2000; LaMotte, Chestertown, MD, USA). Mean telomere length was determined using quantitative real-time PCR as described previously [55]. This method measures the average ratio between the telomere repeat copy number and that of a single-copy gene (36B4; T/S ratio) in each sample. The T/S ratio is proportional to the average telomere length, and the relative telomere length can therefore be calculated quantitatively. Relative telomere length was calculated from T/S ratio = $2^{-\Delta Ct}$, where $\Delta Ct = Ct_{telomere} - Ct_{36B4}$. Primers specific for telomeres: (1: 5′-GGTTTTTGAGGGTGAGGGTGAGGGTGAGGG TGAGGGT-3′; 2: 5′-TCCCGACTATCCCTATCCCTA TCCCTATCCCTATCCCTA-3′) Primers specific for the single-copy gene: (36B4u: 5′-CAGCAAGTGGGAAGG TGTAATCC-3′; 36B4d: 5′-CCCATTCTATCATCAAC GGGTACAA-3′).

Primers design

We obtained FASTA format sequences from the University of California Santa Cruz (UCSC) Genome Browser (http://genome.ucsc.edu/) and used Primer6 software to design primers. Then we used Nucleotide Basic Local Alignment Search Tool (BLAST) (https://blast.ncbi.nlm.nih.gov/Blast.cgi) to check the primer pairs we selected.

RNA interference

siRNAs targeting *RP11-670E13.6*, *HNRNPH1*, and *HNRNPF*; siRNA NC; miRNA mimics; mimics NC; miRNA inhibitor; and inhibitor NC were purchased from RiboBio. The effective interference sequences were all selected by RT-qPCR for the best gene silencing effect and then used for subsequent experiments. The sequences are listed in Supplementary Table 2. For transient transfection, primary HDFs in passages 3–8 were plated in growth medium. After the cells reached 30–50% confluence, cells were transfected with siRNA , miRNA inhibitor or miRNA mimics using a riboFECTTM CP Transfection Kit (RiboBio). Forty-eight hours after transfection, the cells were irradiated with UVB as described above and further cultured with complete medium for 24 h before conducting subsequent experiments.

Detection of SOD and CAT activity

After cells were lysed, the total protein was extracted to detect the activity of SOD and CAT by using the Total Superoxide Dismutase Assay Kit-WST ® (Dojindo, Japan) and the Total Catalase Analysis Assay Kit (Solarbio Science & Technology Co. ltd., Beijing) according to the manufacturers' instructions.

FISH

In situ hybridization was performed with a FISH Kit (RiboBio). HDFs were briefly rinsed in phosphate-buffered saline (PBS) and fixed in 4% formaldehyde for 10 min. The cells were then permeabilized in PBS containing 0.5% Triton X-100 at 4°C for 5 min, washed with PBS three times for 5 min, and prehybridizated at 37°C for 30 min before hybridization. Next, anti-*RP11-670E13.6*, anti-*U6*, and anti-18S oligodeoxynucleotide probes were added in hybridization solution at 37°C overnight in the dark. The next day, the cells were counterstained with 4′,6-diamidino-2-phenylindole and imaged using a Leica DFC300 FX microscope (Germany).

Isolation of nuclear and cytoplasmic RNAs

Cells were collected and washed with ice-cold PBS twice. After centrifugation 1000 g for 5 min, supernatants were removed. Cell pellets were

resuspended in 0.5% v/v NP40-PBS by pipetting gently. After centrifugation 1000 g for 5min, the supernatant was collected as the cytoplasmic fraction and the pellet was washed in ice-cold 0.5% NP40-PBS for two times. The supernatant was discarded and the pellet was nucleus. RNA was extracted by TRIzol reagent following the manufacturer's protocol.

Measurement of intracellular ROS

N-acetyl-L-cysteine (NAC), the ROS scavenger, was purchased from Beyotime (Shanghai, China). Cells were pre-treated with NAC (10 mM) for 1 h before UVB irradiation and were cultured continuously in complete culture medium with NAC (10 mM) after UVB irradiation. Twenty-four hours or eight hours after UVB irradiation, cells were incubated in serum-free medium with 10 mM DCFH-DA (Applygen, Beijing, China) for 30 min at 37°C, according to the manufacturer's protocol, and then washed three times with DMEM. Images were captured using a fluorescence microscope (Nikon Eclipse TS100). The green fluorescence were measured to evaluate the levels of intracellular ROS using Image J software version 1.8.0 (National Institutes of Health, USA).

Immunofluorescence

Cells were seeded and fixed on 12 × 12-mm glass slides. For intracellular staining, the cells were fixed with 4% paraformaldehyde for 30 min, permeabilized with 0.2% Triton X-100 for 10 min, and then blocked with 4% bovine serum albumin (BSA) at 37°C for 30 min. After washing with phosphate-buffered saline (PBS) for 3 × 5 min, the cells were incubated with gamma H2A.X (ab81299,1:50) overnight at 4°C and then incubated with the specified secondary antibodies (Alexa Fluor® 488-conjugated goat anti-rabbit IgG, 1/100) for 2 h. Nuclei were counterstained with 4,6-diami-dino-2-phenylindole for 10 min at room temperature. Fluorescent images were obtained using a Leica DFC300 FX microscope (Germany). Digital microphotographs of 20 fields randomly selected from both UVB-irradiated HDFs transfected with control siRNA and *RP11-670E13.6* siRNA were obtained, and images of each HDF were captured. The average area of γH2AX foci per cell for each treatment was automatically calculated using Image J software version 1.8.0 (National Institutes of Health, USA).

Cell cycle analysis

After siRNA or miRNA inhibitor transfection for 48 h, HDFs were trypsinized, washed with PBS and fixed in 70% ethanol at -20 °C overnight. The cells were then treated with 50 mg/l RNase (Sigma-Aldrich, St. Louis, MO, USA) and stained with 50 mg/l propidium iodide (Sigma) in the dark at 37°C for 30 min. The cell cycle was analyzed using flow cytometry (Cytomics FC500; Beckman Coulter, Fullerton CA, USA).

Annexin V and PI staining and flow cytometry

The percentages of early and late apoptotic cells in HDFs transfected with treatment of *miR-663a* mimics and miRNA mimics control were measured using the APC Annexin V and PI apoptosis detection kit and flow cytometry, according to the manufacturer's instruction. HDFs were seeded at a density of 5×10^4 cells/well in 6-well plates. Cells were collected by centrifuging at 1,000 rpm for 5 min and washed twice with PBS at day 3 after transfection. The cells were simultaneously stained with Annexin V-FITC and the non-vital dye PI, which allowed the identification of intact cells, early apoptotic cells, and late apoptotic cells.

Western blot analysis

Nuclear-cytoplasmic fractionation was conducted using an NE-PER Nuclear and Cytoplasmic Extraction Reagents kit (Thermo Fisher Scientific Inc., Rockford, IL, USA). Proteins were extracted from cells and quantified using a BCA protein assay kit (Bio-Rad, Hercules, CA, USA). Equal amounts of protein were separated using 10% sodium dodecyl sulfate-polyacrylamide gels (Bio-Rad) and then transferred onto polyvinylidene difluoride membranes (Millipore, Billerica, MA, USA). After blocking in a solution of 5% nonfat dry milk diluted in Tris-buffered saline, the membranes were incubated with primary antibodies overnight at 4°C. After incubation with corresponding secondary antibodies conjugated to horseradish peroxidase, the signals of the membranes were detected using an enhanced chemiluminescence western blotting substrate (Pierce, Rockford, IL, USA). The band intensities from western blotting and normalization were carried out using ImageJ (National Institutes of Health, Bethesda, MD, USA). The primary antibodies are listed in Supplementary Table 3.

Comet assays

Neutral comet assays were performed as previously described [56].

Luciferase reporter assays

293T cells were seeded in 12-well plates. After 24 h, the cells were cotransfected with psiCHECK2.0 luciferase reporter vector (Promega, Madison, WI, USA) containing the 3′-UTR fragment of *CDK4,CDK6,CCND1* or pGL3-basic luciferase reporter vector (Promega) containing the

3′-UTR fragment of *RP11-670E13.6*, *Renilla* vector (pRL-TK; Promega), and miRNA mimic NC or miRNA mimic (RiboBio) using Lipofectamine 2000 (Invitrogen). Luciferase activities were measured 48 h after transfection with a Dual-Luciferase Reporter Assay System Kit (Promega) according to the manufacturer's instructions. Firefly luciferase activity was normalized to *Renilla* luciferase activity for each sample.

RNA pull-down assays and mass spectrometry

Biotinylated *RP11-670E13.6* sense and *RP11-670E13.6* antisense were in vitro transcribed using T7 RNA polymerase (Promega) and Biotin RNA Labeling Mix (Roche) and then purified with Quick Spin columns (Roche) according to the manufacturers' instructions. Biotinylated RNAs were mixed and incubated with HDF lysates. Streptavidin agarose beads (Life Technologies, Gaithersburg, MD, USA) were added to each binding reaction, followed by a 1-h incubation period at room temperature. The beads then were washed briefly three times and boiled in sodium dodecyl sulfate buffer. The eluted proteins were detected by standard western blot analysis. *RP11-670E13.6* sense and *RP11-670E13.6* antisense strand protein bands acquired by RNA pull-down assays were excised and examined by mass spectrometry to detect the related proteins that bound directly with *RP11-670E13.6*. The procedure was carried out according to standard protocols, as described previously.

RIP

RIP assays were performed according to the guidelines in the Magna RIP RNA-Binding Protein Immunoprecipitation Kit (Millipore). Briefly, cells were lysed in lysis buffer containing a protease inhibitor cocktail and RNase inhibitor. Magnetic beads were pre-incubated with an anti-flag antibody or anti-rabbit IgG for 30 min at room temperature, and lysates were immunoprecipitated with beads at 4°C overnight. RNA was purified from RNA-protein complexes and analyzed by qRT-PCR. Total RNAs and positive/negative controls were also assayed to demonstrate that the detected signals were from RNAs that bound specifically to hnRNP F/H.

Detection of cell viability

Cells were seeded into 96-well plates in 100 μL medium per well. After different treatments, HDFs were mixed with 10 μL CCK-8 reagent (Dojindo, Kamimashiki-gun, Kumamoto, Japan) per well in normal culture medium for 2 h, and the absorbance at 450 nm was measured with an enzyme mark instrument (Thermo Fisher Scientific).
