# Supplementary material for: LncRNA RP11-670E13.6, interacted with hnRNPH, delays cellular senescence by sponging microRNA-663a in UVB damaged dermal fibroblasts
Source: Aging (Albany NY). 2019 Aug 23;11(16):5992–6013. doi: 10.18632/aging.102159 (PMC6738423; doi:10.18632/aging.102159)
Supplement: Supplementary Figures [file aging-11-102159-s003.pdf]

SUPPLEMENTARY FIGURES

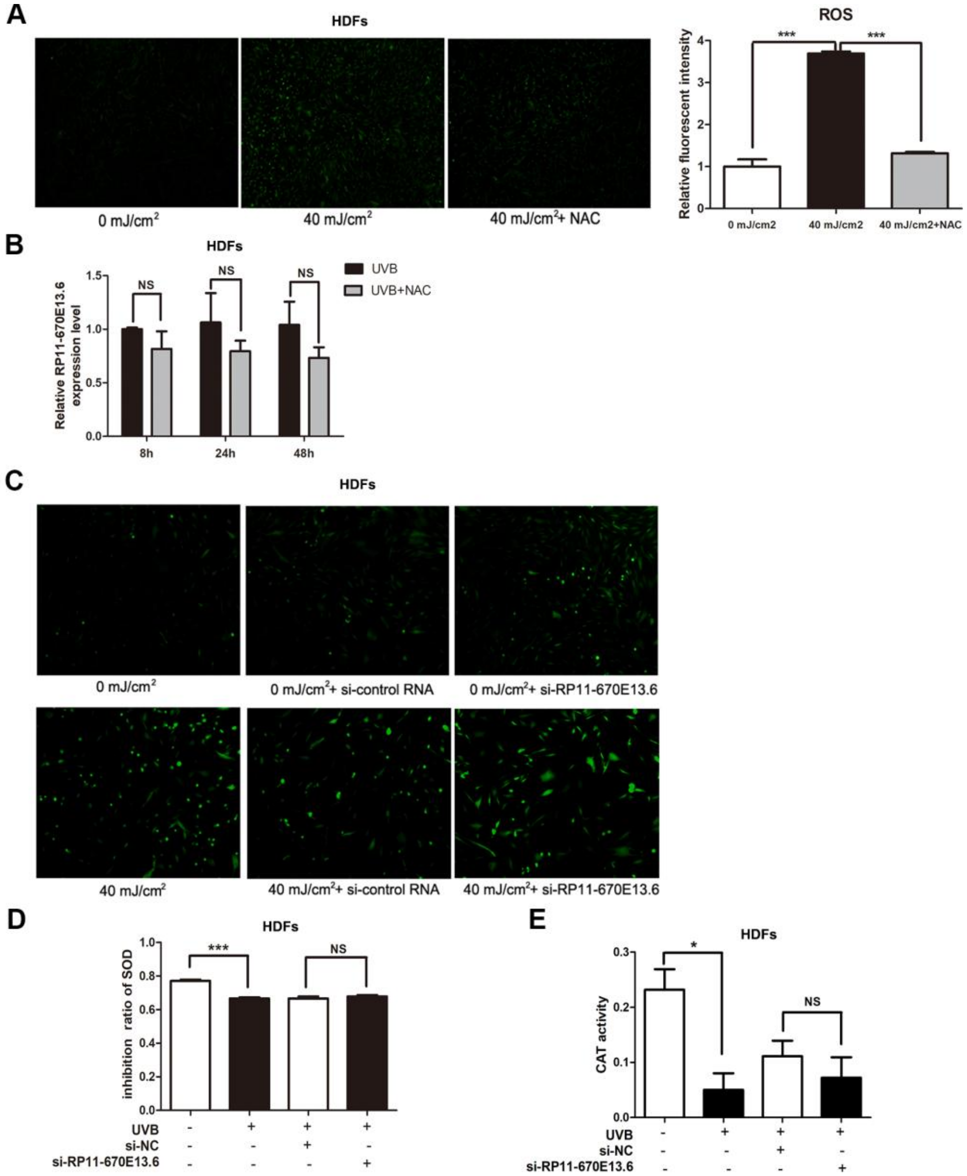

Supplementary Figure 1. (**A**) 24 h after exposure to 40 mJ/cm^2 UVB, ROS content (magnification, 40×) in the UVB irradiation group was increased compared with that in the control group, and NAC (10 mM) caused a reduction in UVB-induced ROS generation. (**B**) At 24 h after exposure to 40 mJ/cm^2 UVB, NAC had no significant effect on UVB-induced upregulation of *RP11-670E13.6.* (**C**) ROS contents were not influenced in *RP11-670E13.6*-depleted HDFs compared with that in the control group (magnification, 40×). (**D**, **E**) Activities of antioxidant enzymes SOD and CAT. CAT, catalase; NS, not significant; SOD, superoxide dismutase.

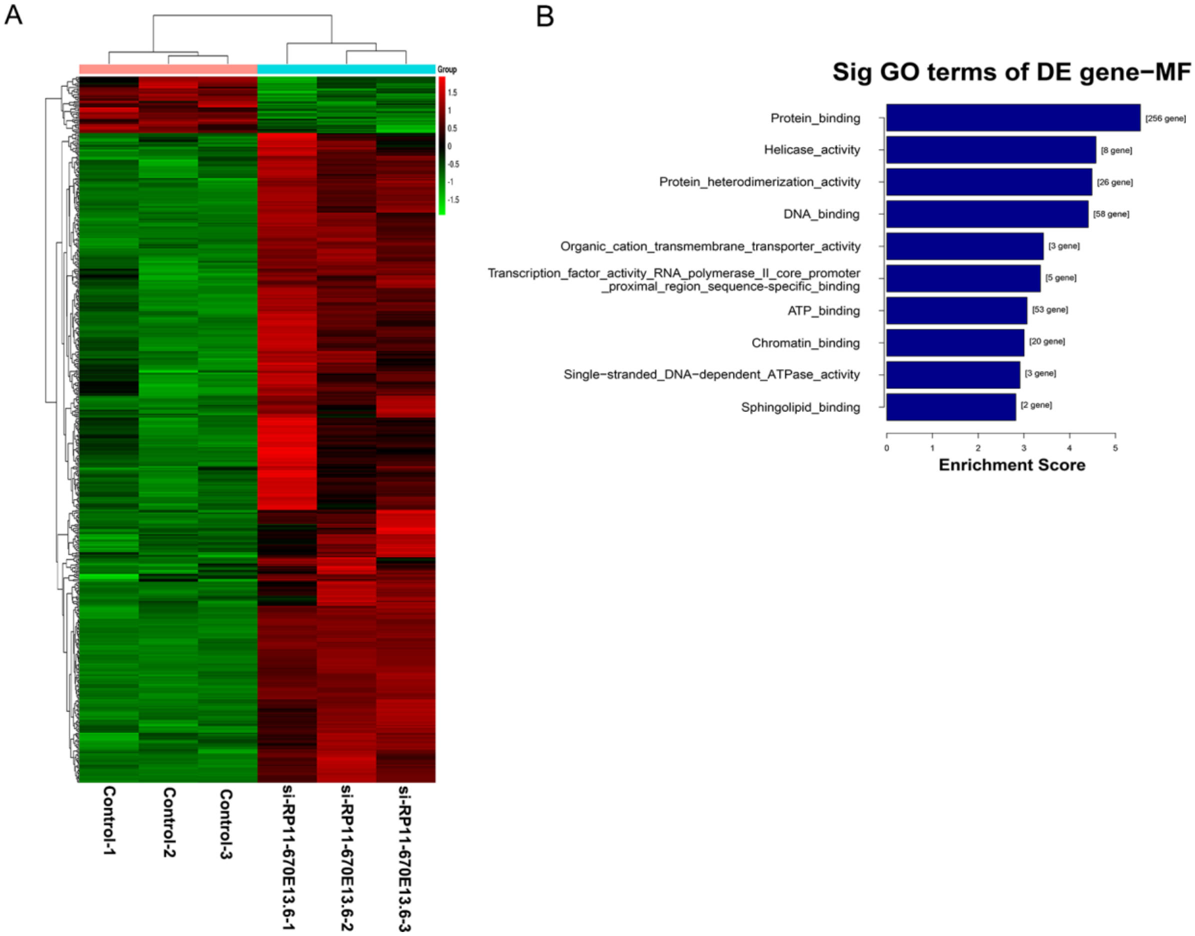

Supplementary Figure 2. (**A**) HDFs were transfected with *RP11-670E13.6* or control siRNA. Forty-eight hours after transfection, whole-transcriptome analysis was performed with RNA-seq. Heatmap showing the differentially expressed genes after *RP11-670E13.6* knockdown ($P < 0.05$, FC log2 > 1.5). (**B**) Top significant molecular functions for genes whose transcript levels were increased in *RP11-670E13.6*-depleted HDFs.

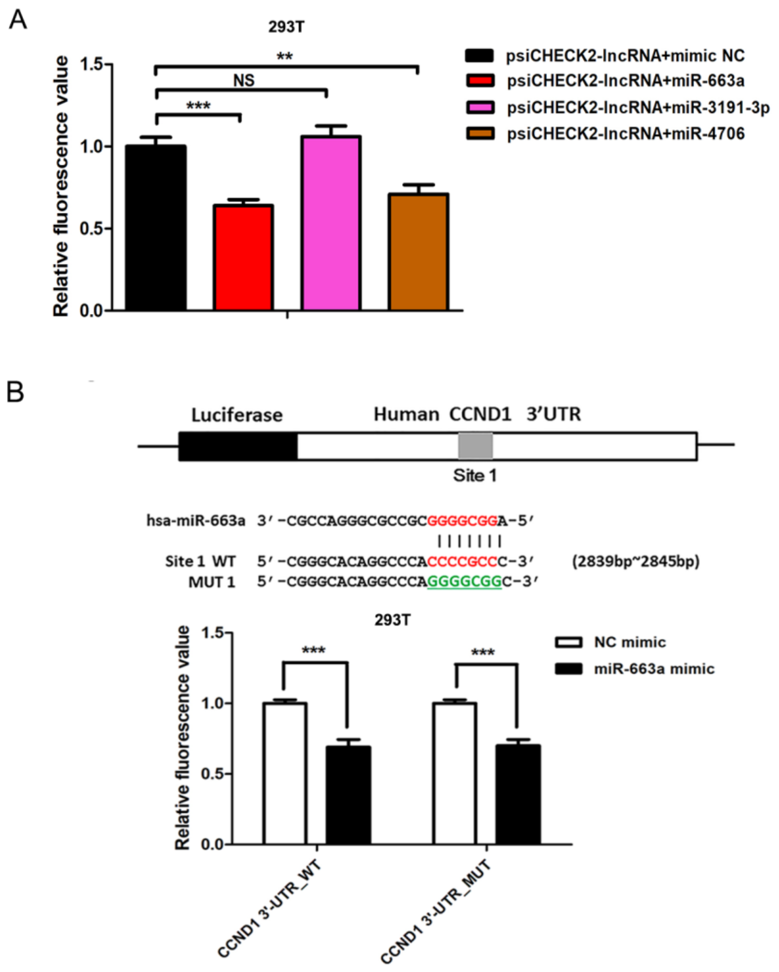

Supplementary Figure 3. (**A**) Luciferase assays showed a significant decrease in luciferase activities after cotransfection of the *RP11-670E13.6* expression vector and miRNA mimics. (**B**) Putative binding site of *miR-663a* in the 3′-UTR of *CCND1* and the sites of target mutagenesis are indicated. Luciferase activity in HDFs, demonstrating the effects of *miR-663a* on the expression of *CCND1*. *P* values were determined by Student's *t*-tests. **P* < 0.05; ***P* < 0.01; and ****P* < 0.001.
