# Supplementary material for: LncRNA RP11-670E13.6, interacted with hnRNPH, delays cellular senescence by sponging microRNA-663a in UVB damaged dermal fibroblasts
Source: Aging (Albany NY). 2019 Aug 23;11(16):5992–6013. doi: 10.18632/aging.102159 (PMC6738423; doi:10.18632/aging.102159)
Supplement: Supplementary Tables [file aging-11-102159-s002.pdf]

SUPPLEMENTARY TABLES

Supplementary Table 1. Primers used for qRT-PCR.

Gene	Forward primer(5′-3′)	Reverse primer(3′-5′)
GAPDH	GGGAAACTGTGGCGTGAT	GAGTGGGTGTCGCTGTTGA
RP11-670E13.6	CACTCTGCGGATGAGGAAG	AGATGAGTGCTGGGAAGGAG
CCN2/CTGF	GTTTGGCCCAGACCCAACT	GGAACAGGCGCTCCACTCT
ATM	TGGATCCAGCTATTTGGTTTGA	CCAAGTATGTAACCAACAATAGAAGAAG
ATR	TGTCTGTACTCTTCACGGCATGTT	AAGAGGTCCACATGTCCGTGTT
CHK1	GGTGAATATAGTGCTGCTATGTTGACA	TTGGATAAACAGGGAAGTGAACAC
MDM2	GGCAGGGGAGAGTGATACAGA	GAAGCCAATTCTCACGAAGGG
GADD45A	GAGAGCAGAAGACCGAAAGGA	CAGTGATCGTGCGCTGACT
U6	CTCGCTTCGGCAGCACA	AACGCTTCACGAATTTGCGT
CDK4	GAG GCGACTGGAGGCTTTT	GGATGTGGCACAGACGTCC
CDK6	TCAGGTTGTTTGATGTGTGC	TCCTTTATGGTTTCAGTGGG
CCND1	GCTGCGAAGTGGAAACCATC	CCTCCTTCTGCACACATTTGAA
HNRNPH1	TGGCTATAATGATGGCTATGG	GTGTCCTGTTGTGCTCTG
HNRNPF	AACTGCCTCTGCTACAAC	ACACTTCTGGATGGTAATGA

Supplementary Table 2. Interference sequences.

Gene	The interference sequences
h-RP11-670E13.6	TAGCAGCGCTGGTTATATT<br>CCACTCTGCGGATGAGGAA<br>GCACTCATCTGAGACCAGA<br>TTAGAGCATCCTCGCGACCA<br>TCATCTGAGACCAGAGGTGT<br>CCACTCTGCGGATGAGGAAG
h-HNRNPH1	GGTCCAAATAGTCCTGACA<br>GATCCACCACGAAAGCTTA<br>GTTCGCAACTCATGAAGAT
h-HNRNPF	GGAAGTTAGGTCATACTCA<br>ACCGGTACATTGAGGTGTT<br>AAGCGACCGAGAACGACAT

Supplementary Table 3. Primary antibody information.

Antibody	WB	Product code	Company
γH2AX	1:2000	ab81299	abcam
Cdk4	1:1000	ab137675	abcam
Cdk6	1:2000	ab151247	abcam
CyclinD1	1:2000	ab40754	abcam
ATM	1ug/ml	ab82512	abcam
hnRNPH/F	1:1000	ab10689	abcam
β-actin	1:5000	ab8226	abcam

Supplementary Table 4. Primers used for PCR amplification.

Gene	Primer sequence
CDK4- 3′UTR	F GCATGCGATCGCCCTGATTGGGCTGCCTCCAGA
	R AATGCGGCCGCTAGGCCCTGTAATTTAACCA
CDK6- 3′UTR	F GGCGCTCGAGTCCTTAGCACAGCACCACAG
	R AATGC GGCCGCTCCAGGCATATCTTTCACCA
CCND1- 3′UTR	F GGCGCTCGAGCCTGTGATGCTGGGCACTT
	R AATGCGGCCGCCATGTTGGTGCTGGGAAGG
RP11-670E13.6	F (EcoRI) AAAAGAATTC GAGCTGGCGAAGGTCG
	R (NotI) AAAGCGGCCGC TGTGGTTTAACAGTTCCTTTTTATT
